# Physical match demands across different playing positions during transitional play and high-pressure activities in elite soccer

**DOI:** 10.5114/biolsport.2024.131815

**Published:** 2023-04-10

**Authors:** Lukasz Bortnik, Stewart Bruce-Low, Joost Burger, Jill Alexander, Damian Harper, Ryland Morgans, Christopher Carling, Kevin McDaid, David Rhodes

**Affiliations:** 1 Football Performance Hub, Institute of Coaching and Performance (ICaP), School of Sport and Health Sciences, University of Central Lancashire, Preston, United Kingdom; 2 Performance & Sport Science Department, Hapoel Beer Sheva FC, Israel; 3 Department of Applied Sport and Exercise Science, University of East London, United Kingdom; 4 Vrije Universiteit Amsterdam, Netherlands; 5 France Football Federation, Paris, France; 6 Dundalk Institute of Technology, Dundalk, Louth, Ireland; 7 Human Performance Department, Burnley FC, United Kingdom

**Keywords:** Soccer, Transitions, High pressure, Peak demands, Worst-case-scenario, Positions

## Abstract

This study explored physical match demands across different playing positions during transitional play, to inform the need for position-specific training interventions. Data was collected using 10 Hz GPS units from 10 competitive matches including 23 elite soccer players of the 1^st^ Polish Division (Ekstraklasa) in season 2020–21. A total of 4249 positional observations were made; center backs (n = 884), full backs (n = 972), central defensive midfielders (n = 236), central attacking midfielders (n = 270), central midfielders (n = 578), wingers (n = 778), and attackers (n = 531). Match data reflected distances covered per minute (m · min^−1^): total distance (TD), high-speed running distance (HSRD, > 19.8 km · h^−1^), sprint distance (SD, > 25.2 km · h^−1^), and the frequency of high-intensity accelerations and decelerations (A+D, > 3 m · s^−2^; n · min^−1^). Total absolute sprint distance (SD, > 25.2 km · h^−1^) and total relative sprint distance (Rel B5) were also quantified. A univariate analysis of variance revealed position-specific differences. Significant effects of position were found for all analysed metrics during transitional play (large ESs; p <.001). Central attacking midfielders displayed higher TD (m · min^−1^), fullbacks covered highest SD (m · min^−1^) and wingers achieved the highest A+D (n · min^−1^) (p ≤ 0.05). Centre backs displayed the lowest physical outputs when compared to all other positions, except in A+D (n · min^−1^) during defensive transitions (p ≤ 0.05). Attackers displayed the highest physical metrics during high pressure activities (p ≤ 0.05). Coaches should carefully consider positional transitional demands to better inform training design. With specific attention paid to drills that replicate game play.

## INTRODUCTION

Soccer match play is characterized by short linear high-velocity actions and multidirectional accelerations and decelerations, mixed with lower intensity recovery breaks of longer duration [1]. With the most decisive and crucial moments involving explosive actions (high-speed running and sprinting) combined with varied technical skills [1, 2, 3]. Thus, emphasizing the need to carefully consider the inclusion of physical, technical, and tactical demands within modern training design, optimally preparing players for high-intensity passages experienced within game play [4]. Modern microtechnology (i.e., Global Positioning System) has become commonplace in professional soccer allowing quantification of team and individual external loads. GPS has been shown to be a valid and reliable method of quantifying team and positional demands, albeit commonly utilized to display maximum and average outputs over the duration of a session or game [5]. This approach fails to quantify the isolated physical demands experienced by players during transitional play, where short bouts of high-intensity actions are performed [6].

Although average session data provides valuable information about the volume of the activity, it does not represent fluctuations in physical, technical, and tactical intensity. Thus, potentially underestimating the most demanding periods within a modern game and importantly providing no accurate differentiation of these high-intensity passages in relation to individual positional demands [7]. Literature details that distances covered at varied intensities would differ between positional groups [1]. Many studies have reported that midfield players cover the most total distance, whilst wide players (fullbacks, wingers) and forwards complete more high intensity running [8–9]. Research surrounding peak intensity passages, with specific reference to technical and tactical activities, is limited in elite soccer [10, 11, 12]. Understanding positional differences during crucial high-intensity transitional periods, would provide definitive insight of how to best prepare the players for game demands. Allowing practitioners to contextualize the physical outputs required in specific drills, ensuring the physical demand complements the tactical requirements placed on players by the coaches [13, 14, 15].

Offensive (defense-to-attack) and defensive (attack-to-defense) transitions represent key moments of play in soccer, that directly influence match outcome [10, 16]. During these critical phases of match-play goal scoring opportunities are created, with teams taking risks to capatilise quickly on the opposition conceding possession [16]. The main objective of offensive transitional activities (counter-attack and fast attack) is to achieve numerical superiority in the opponent’s half by performing high-speed actions. Contrastingly, the defensive transition is characterized by re-shaping an imbalanced defense to effectively slow down the opposition attack [17]. It is noteworthy that elite soccer teams attack at a very high tempo that lasts below 20 seconds [14, 18]. Counterattacks have been shown to be the most productive playing style for scoring goals [19] often influenced by a number of contextual factors [20]. High-pressure activities were identified as an effective strategy to score goals and create more goal scoring opportunities [17]. These actions have been associated with high fitness levels and high physical demands [21]. TA’s have been well described in the literature and directly connected with technical and tactical activities occurring concurrently with other physically stressful moments [17]. Moreover, TA’s have a deep context within since it encompasses moments when the ball is in and out of possession (offense and defense, respectively) as well as describing periods of high tactical component such as offensive activities (counter-attack and fast attack) and defensive actions (attack-to-defense transition and high pressure).

It is hypothesized that physical outputs would differ across playing positions during transitions. Moreover, it is suggested that defensive players (center backs, fullbacks, and central defensive midfielders) would be exposed to higher physical stress during defensive phases, with offensive players (wingers, central attacking midfielders, and attackers) within attacking moments of game play. Wide players (fullbacks and wingers) would accumulate greater high-velocity distances during transitional activities. Therefore, the aims of the present study were, 1) investigate physical match demands across different playing positions during transitional play in elite soccer; and 2) identify positional differences in different relative and absolute physical metrics (total distance, high-speed distance, sprint distance, and accelerations/decelerations) during specific short-duration actions such as offensive transitions (counter-attacks), defensive transitions, fast attacks and high-pressure activities.

## MATERIALS AND METHODS

### Participants

Twenty-three male elite soccer players participated in the present study. Players belonged to a leading team of the 1^st^ Polish Division (Ekstraklasa) in season 2020–21 and they were classified according to playing position, resulting in the following number per position: center backs (*n* = 4), full backs (*n* = 5), central defensive midfielders (*n* = 2), central attacking midfielders (*n* = 2), central midfielders (*n* = 2), wingers (*n* = 5), and attackers (*n* = 3). Only players who completed at least 60 minutes were included in this investigation, due to pacing strategies utilized by substitutes potentially generating higher outputs than starting players [7, 13]. Players received all information about the project protocol and provided informed consent for the use of match data, in accordance with the Helsinki Declaration. To ensure player confidentiality, all data was anonymised prior to analysis. Ethical approval was provided by the University of Central Lancashire (HEALTH 0104).

### Procedures

Data from a total of ten official games including one UEFA CL qualifier and nine Polish domestic league (Ekstraklasa) games (6 wins, 1 draw, and 3 losses) were collected and analysed in the 2020–2021 season. The CL qualifier was included due the opposing team competing at a comparable level, in terms of budget and league position, within another eastern European country. A low number of analysed matches were observed due to a manager change, could have skewed the results due to tactical differences. A total of 4249 individual observations were extracted including 1164 offensive transitions, 1269 defensive transitions, 1120 fast attacks, and 696 high pressure activities. The following number of observations per position were recorded: centre backs (*n* = 884), full backs (*n* = 972), central defensive midfielders (*n* = 236), central attacking midfielders (*n* = 270), central midfielders (*n* = 578), wingers (*n* = 778), and attackers (*n* = 531). The activity profile of players was captured by MEMS (10 Hz; Vector S7, Catapult Sports, Melbourne, Australia). The GPS model used in this study was worn in each game between the scapulae and contained within the playing jersey inside a mini pocket and thus not affecting mobility of the upper limbs. The players wore these devices in training and games and thus, were familiar with the entire procedure. To get an optimal connection to the satellites, the devices were turned on 15 mins before the start of the game. In accordance with previous guidelines for acceptable GPS coverage, each data was screened for satellite coverage and horizontal dilution of precision (HDOP) using an inclusion criterion of > 6 satellites and ≤ 1.0 respectively [22]. To avoid the inter-unit variations, each player used the same device in matches. The accuracy of this technology has been previously presented in detail [5, 23].

All variables selected for this study have been previously utilised within research [4, 13, 14, 24]. Displaying ICC values between 0.77 (95% CI: 0.62–0.89) (very large) to 1.0 (95% CI: 0.99–1.0) (nearly perfect) for the measurement of distances at different velocity bands, indicating excellent intra-unit reliability [5, 23]. For mechanical load, acceptable reliability was found for accelerations (CV = 1.4 ± 1.5% to 4.2 ± 1.5%), and acceptable to poor reliability for decelerations (CV = 2.5 ± 1.5% to 10.9 ± 1.5%) [5, 23]. These variables reflected absolute distances covered per minute (m · min^−1^) in the following categories: total distance (TD), highspeed running distance (HSRD, > 19.8 km · h^−1^), sprint distance (SD, > 25.2 km · h^−1^), as well as the number of high-intensity accelerations and decelerations (A+D, > 3 m · s^−2^; n · min^−1^). Also, the variables reflected total absolute sprint distance (SD, > 25.2 km · h^−1^) and total relative sprint distance (Rel B5), which was proposed previously by other authors to represent the functional limits of endurance and sprint locomotor capacities [25]. Relative sprint distance (Rel B5) was set above maximum aerobic speed (MAS) and plus 30% for anaerobic speed reserve (ASR) [25]. An incremental running treadmill test was conducted by the club physiologist to measure VO_2max_ and MAS. The test was performed in the gym environment with a normal ambient temperature and took place on a mechanical treadmill (Technogym, Italy). It began with an initial speed of 10 km/h^−1^ and each stage was increases by 1.5 km/h^−1^. Five stages were set. Each stage lasted 4 minutes and it was separated by 1 minute passive break. The inclination was set at 1.5%. Polar heart rate monitors (Polar, Norway) and Polar M400 are used to record HR data. Expired gases were analysed breath-bybreath using an online automated gas analysis system (MetaLyzer® 3b-R2; Cortex Biophysik Gmbh, Leipzig, Germany) and accompanying software (MetaSoft® 3). Maximum oxygen uptake (VO_2max_) was defined as the highest 15-s average oxygen uptake. Velocity (km/h^−1^) during the maximum oxygen uptake (VO_2max_) was recorded and set as the maximum aerobic speed (MAS).

TA’s were identified and manually created by the club’s analysis team in the Catapult Vision video analysis system (Catapult Sports Ltd, Melbourne, Australia) after each match was completed. Phases of play were classified into the following categories: offensive transition (counterattack) (OT), defensive transition (opposition’s counterattack) (DT), fast attack (FA), and high pressure (HP), which were previously defined and analysed by others [14, 17]. Offensive transitions and fast attacks ended when the following occurred: a) loss of ball possession (ball of out play and/or cleared by opposition), b) shot on goal, c) decreased tempo of attack due to an effective defense by opposition. Defensive transitions ended if the following events took place: a) possession re-gained, b) ball out of play, c) shot on goal, d) decreased tempo of attack due to an effective defense by opposition. High-pressure activities ended when: a) possession re-gained, b) ball kicked forward by team in possession, c) ball out of play.

The observational methodology REOFUT theoretical framework was used to identify these actions by one member of the club’s analysis team [26] and checked by an independent analyst working for the club’s software supplier. The analysis procedure using the REO-FUT instrument involved in the current body of work was part of the club’s analysis protocols implemented daily by the analysis team.

Previous literature has demonstrated good to high intra- and inter-observer reliability of this analysis method [17, 27]. Inter-observer and intra-observer analysis indicated adequate levels of reliability for the phases analysed in the study based on Cohen’s Kappa calculations (OT: 0.819, 0.964; DT: 0.815, 0.818; FA: 0.959, 0.962; HP: 0.775, 0.896, for inter- and intra-observer reliability, respectively). High levels of objectivity are detailed for OT, DT and FA, and reduced objectivity for HP [28]. Both analysts had 8 years plus experience of working as part of an analysis team within elite football. Prior to the analysis of the ten games reliability and objectivity checks between the two analysts were completed. Both analysts were asked to utilize the RE-OFUT methodology to analyse two 11v11 training matches which were separated by 7 days, with each game consisting of two 30-minute halves. Data from the Catapult vision software was then downloaded and integrated into the manufacturer’s software package (*Open-field*, version 3.2.0) and finally exported into Microsoft Excel (Microsoft Corporation, USA) to make additional calculations for each transitional play. The transition mean average for selected metrics was calculated as the sum of all TA’s and divided by their number. For each of the observed transition periods within the two 11v11 games an agreement of > 85% was noted between the two analysts.

### Statistical analysis

A descriptive analysis was used, and the results are shown as mean ± standard deviation (SD). Between-matches coefficient of variation (CV) values were computed for transitions for absolute and relative sprint distance (SD and Rel B5, respectively). Statistical analyses were performed using IBM Statistical Package for the Social Sciences (SPSS, Version 27.0, IBM Corporations, New York, USA) with the statistical significance accepted at the 0.05 level. A univariate analysis of variance (ANOVA) was used to quantify main effects for games, transitions, and positions. Interaction effects were also calculated, and any significant main effects associated with games, transitions, and positions were examined using post hoc pairwise comparisons. The assumptions linked to the statistical model were examined to ensure model adequacy. To establish residual normality for each dependent variable, q-q plots were created using stacked standardised residuals. Scatterplots of the stacked unstandardized and standardised residuals were also used to examine the error of variance associated with the residuals. Mauchly’s test of sphericity was also done for all dependent variables, with a Greenhouse Geisser correction applied if the test was significant. *A priori* power calculations were conducted using familiarisation trials and pilot data completed by participants matching the criteria described above. Across all isokinetic and stabilomtery measures considered within the thesis, a sample size ≥ 14 players was required to evaluate the interactions associated with all independent variables (for statistical power > 0.8; *p* < 0.05). Partial eta squared (η^2^) were computed to estimate effect sizes for all significant main effects and interactions. Partial eta squared was classified as small (0.01–0.059), moderate (0.06–0.137), and large (> 0.138) as suggested previously [29].

## RESULTS

**FIG. 1:**
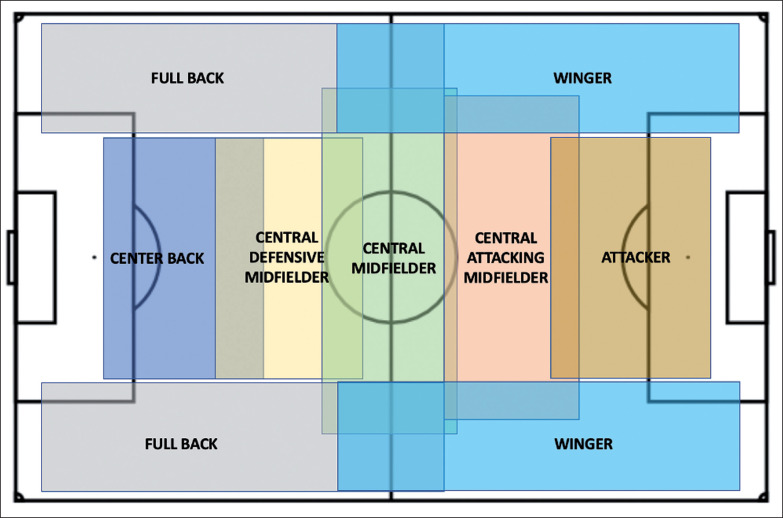
General and specialised tactical roles based on game analyses. Adapted from Aalbert and Van Haaren [30].

Figure 1 depicts how players were assigned to their general and specialized playing position in accordance with their tactical roles and responsibilities [30].

Table 1 highlights the mean for selected physical variables for each playing position during transitions that occurred across all 10 analysed matches.

**TABLE 1:** Mean ± SD for TD (m · min^−1^), high-speed running distance (HSRD) (m · min^−1^), sprint distance (m · min^−1^), accelerations and decelerations (A+D) (n · min^−1^) for each playing position CB (Center Back), FB (Full Back), CDM (Central Defensive Midfielder), CM (Central Midfielder), CAM (Central Attacking Midfielder), W (Winger), and A (Attacker) during transitional activities across 10 official matches.

	All (n = 4249)	CB (n = 884)	FB (n = 972)	CDM (n = 236)	CM (n = 578)	CAM (n = 270)	W (n = 778)	A (n = 531)
**Total distance [m · min^−1^]**	206.2 ± 19.0	166.8 ± 63.7^b,c,d,e,f,g^	204.2 ± 81.4^a,e^	214.9 ± 59.2^a,e^	212.3 ± 66.6^a,e^	234.4 ± 58.6^a,b,c,d,f,g^	211.7 ± 72.6^a,e^	203.6 ± 73.7^a,e^

**HSRD [m · min^−1^]**	79.8 ± 11.6	21.7 ± 57.1^b,c,d,e,f,g^	61.2 ± 94.2^a,c,d^	44.5 ± 70.4^a,b,e,f^	45.6 ± 81.9^a,b,e,f^	67.4 ± 87.2^a,c,d^	63.4 ± 87.7^a,c,d^	53.0 ± 77.3^a^

**Sprint distance [m · min^−1^]**	13.2 ± 7.0	5.0 ± 26.7^b,d,f^	24.2 ± 62.2^a,c,d,e,g^	5.5 ± 26.2^b,f^	12.9 ± 42.8^a,b^	13.5 ± 38.8^b^	20.0 ± 54.1^a,c,g^	10.9 ± 37.2^b,f^

**A+D [m · min^−1^]**	1.1 ± 0.3	0.7 ± 2.3^b,d,f,g^	1.1 ± 2.9^a,f^	0.9 ± 2.7^f^	1.1 ± 3.0^a,f^	1.1 ± 2.6^f^	1.7 ± 3.5^a,b,c,d,e^	1.3 ± 2.9^a^

Note: Significant differences

a CB,

b FB,

c CDM,

d CM,

e CAM,

f W, and

g A (p < 0.05).

Statistically significant interactions between game, transition and position were found for TD (m · min^−1^) and HSRD (m · min^−1^) (TD: *F*_(131,4010)_ = 1.506, *p* <.001, partial η^2^ =.047; HSRD: *F*_(131,4010)_ = 1.223, *p* =.045, partial η^2^ =.038). A significant interaction between transition and position were identified for SD (m · min^−1^), A+D (n · min^−1^), Absolute SD and Relative SD (Rel B5) (SD: *F*_(18,4010)_ = 6.385, *p* <.001, partial η^2^ =.028; A+D: *F*_(18,4010)_ = 9.227, *p* <.001, partial η^2^ =.040; Absolute SD: *F*_(18,4010)_ = 6.211, *p* <.001, partial η^2^ =.027; Relative SD (Rel B5) *F*_(18,4010)_ = 16.337, *p* <.001, partial η^2^ =.068). Removal of the CL game for analysis indicated negligible change to the observed interactions for game, transition and position (TD: *F*_(131,4010)_ = 1.514, *p* <.001, partial η^2^ =.044 and HSRD: *F*_(131,4010)_ = 1.293, *p* =.02, partial η^2^ =.038) and for transition and position (Rel B5) (SD: *F*_(18,4010)_ = 6.672, *p* <.001, partial η^2^ =.031; A+D: *F*_(18,4010)_ = 9.081, *p* <.001, partial η^2^ =.041; Absolute SD: *F*_(18,4010)_ = 6.826, *p* <.001, partial η^2^ =.031; Relative SD (Rel B5) *F*_(18,4010)_ = 16.907, *p* <.001, partial η^2^ =.074). There was a statistically significant main effect of transitions on duration (*F*_(3,456)_ = 9.997, *p* <.0005, partial η^2^ =.062). Offensive transitions (OT) duration was significantly higher than all other transitions (*p* ≤ *0.05*).

Post hoc tests for all ten games revealed that central attacking midfielders (CAM) had higher TD (m · min^−1^) compared to other positional groups (*p* ≤ *0.001*). Wingers (W), fullbacks (FB) and central attacking midfielders (CAM) ran higher HSRD (m · min^−1^) and relative sprint distance (Rel B5) from other positions (*p* ≤ *0.05*). Fullbacks (FB) covered highest SD (m · min^−1^) and Absolute SD (*p* ≤ *0.05*). Wingers (W) had the highest number of A+D (n · min^−1^) compared to other groups (*p* ≤ *0.05*). In contrast, center backs (CB) showed lower TD (m · min^−1^), HSRD (m · min^−1^), A+D (n · min^−1^), and Relative SD (Rel B5) compared to other positions (*p* ≤ *0.05*). Also, they had lower Absolute SD than fullbacks (FB), wingers (W), central midfielders (CM), and central attacking midfielders (CAM) (*p* ≤ *0.05*).

No statistical significance was found between central midfielders (CDM and CM) and wingers (W) for TD (m · min^−1^) (*p* > 0.05). Wingers (W), fullbacks (FB), central attacking midfielders (CAM), and attackers (A) detailed no significant difference positionally for HSRD (m · min^−1^) and relative SD (Rel B5) (*p* > 0.05), however for these metrics these positions were higher than all other positions (p ≤ 0.05). No difference between wingers (W) and fullbacks (FB) for SD (m · min^−1^) and Absolute SD (*p* > 0.05). Center backs (CB) and central defensive midfielders (CDM) had lowest SD (m·min^−1^) (p ≤ 0.05), A+D (n · min^−1^) and Absolute SD, a no statistically significant difference between them (*p > 0.05*). No statistically significant difference was found for Fullbacks (FB), wingers (W), and central attacking midfielders (CAM) for Relative SD (Rel B5) (*p > 0.05*).

Table 2 displays the mean absolute and relative sprint distance for each playing position during all analysed transitions accompanied by match-to-match variability for these physical metrics.

**TABLE 2:** Mean ± SD and match-to-match variability as CV (%) for absolute sprint distance and relative sprint distance for each playing position CB (Center Back), FB (Full Back), CDM (Central Defensive Midfielder), CM (Central Midfielder), CAM (Central Attacking Midfielder), W (Winger), and A (Attacker) during transitional activities across 10 official matches.

	All (n = 4249)	CB (n = 884)	FB (n = 972)	CDM (n = 236)	CM (n = 578)	CAM (n = 270)	W (n = 778)	A (n = 531)	CV (%)
**Absolute sprint distance [m · min^−1^]**	2.2 ± 1.2	0.8 ± 4.1^b,d,e,f^	4.0 ± 10.6^a,c,d,e,g^	1.0 ± 4.7^b,f^	2.1 ± 7.1^a,b^	2.4 ± 6.8^a,b^	3.3 ± 8.6^a,c,f^	1.7 ± 5.6^b,f^	53.8

**Relative sprint distance (Rel B5) [m · min^−1^]**	6.1 ± 2.0	2.6 ± 8.1^b,c,d,e,f,g^	7.2 ± 13.7^a,d^	5.3 ± 9.8^a,f^	5.1 ± 11.5^a,b,e,f^	8.2 ± 13.3^a,c,d,g^	8.2 ± 13.1^a,c,d,g^	5.8 ± 10.2^a,f^	32.7

Note: Significant differences

a CB,

b FB,

c CDM,

d CM,

e CAM,

f W, and

g A (p < 0.05).

**FIG. 2:**
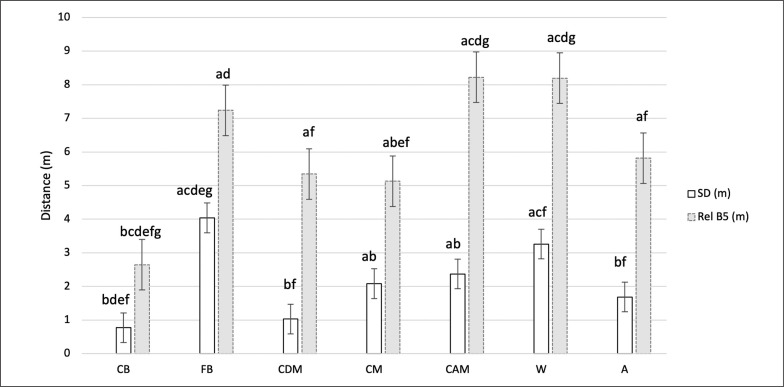
Comparisons between all playing positions CB (Center Back), FB (Full Back), CDM (Central Defensive Midfielder), CM (Central Midfielder), CAM (Central Attacking Midfielder), W (Winger), and A (Attacker) in mean absolute sprint distance – SD (m) (> 25.2 km·h-1) and mean relative sprint distance - Rel B5 (m) (MAS + 30% ASR) during transitional activities (offensive transitions; defensive transitions; fast attacks; high pressures) across 10 official matches. **Significant differences a CB, b FB, c CDM, d CM, e CAM, f W, and g A (p < 0.05).

Figure 2 displays differences in all playing positions for mean absolute and relative sprint distance across all transitions.

**FIG. 3:**
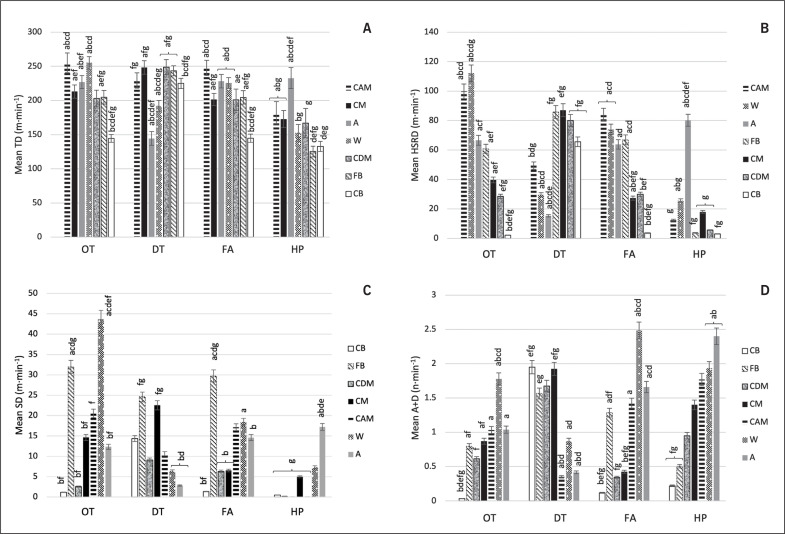
Comparisons between playing positions CB (Center Back), FB (Full Back), CDM (Central Defensive Midfielder), CM (Central Midfielder), CAM (Central Attacking Midfielder), W (Winger), and A (Attacker) in a) mean TD (m·min^−1^) b) mean HSRD (> 19.8 km·h^−1^; m·min^−1^), c) mean SD (> 25.2 km·h^−1^; m·min^−1^), and d) mean A+D (> 3 m·s^−2^; n·min^−1^) during different transitions: OT = Offensive transition; DT = Defensive transition; FA = Fast attack; HP = High pressure. **Significant differences ^a^ CB, ^b^ FB, ^c^ CDM, ^d^ CM, ^e^ CAM, ^f^ W, and ^g^ A (p < 0.05).

Figure 3 depicts differences between all playing positions in mean distances covered per minute (m · min^−1^) for TD, HSRD, SD and mean number of accelerations/decelerations – A+D (n · min^−1^) across all transitional activities.

Analysis of positional effect identified that center backs (CB) had the lowest TD (m · min^−1^), HSRD (m · min^−1^), and A+D (n · min^−1^), a statistically significant difference from all other positions (*p* ≤ *0.05*) during offensive phases such as offensive transitions (OT) and fast attacks (FA). In contrast, they achieved highest A+D (n · min^−1^) during defensive transitions (DT), a statistically significant difference with offensive players such as attackers (A), wingers (W), and central attacking midfielders (CAM) (*p* ≤ *0.05*).

Detailed positional analysis also revealed that SD (m · min^−1^) was highest for fullbacks (FB) (*p* ≤ *0.05*) during defensive transitions (DT) and fast attacks (FA). Similarly, central midfielders (CM) had high SD (m · min^−1^), a statistically significant difference with attackers (A) and wingers (W) (*p* ≤ *0.05*) during defensive transitions (DT). Central attacking midfielders (CAM) revealed significantly higher TD (m · min^−1^), HSRD (m · min^−1^) from other groups (*p* ≤ *0.05*) during offensive transitions (OT) and fast attacks (FA).

Wingers (W) showed statistically higher TD (m · min^−1^), HSRD (m · min^−1^), SD (m · min^−1^), and A+D (n · min^−1^) (*p* ≤ *0.05*) during offensive transitions (OT). Also, this positional group revealed highest output in A+D (n · min^−1^) during fast attacks (FA), a statistically significant difference with other positions (*p* ≤ *0.05*). Attackers (A) had significantly lower TD (m · min^−1^) and HSRD (m · min^−1^) compared to all other positions (*p* ≤ *0.05*) during defensive transitions (DT). However, this positional group had highest TD (m · min^−1^), HSRD (m · min^−1^), and SD (m · min^−1^) from other positions (*p* <.001) and were statistically higher in A+D (n · min^−1^) from all defenders (CB, FB) (*p* ≤ *0.05*) during high pressure (HP). No other significant positional differences were identified for any other physical metrics (*p > 0.05*).

## DISCUSSION

The aims of the present study were to 1) investigate physical match demands across different playing positions during transitional play in elite soccer; and 2) identify positional differences in different relative and absolute physical metrics during offensive transitions (counter-attacks), defensive transitions, fast attacks and high-pressure activities. The main findings of our study indicated that physical performance during transitions varied between different playing positions. The activity profile of elite soccer players within 90-min match play has been found to be highly dependent on the tactical role and playing position [1, 31–32]. Previous research has identified midfielders covering the longest average distance followed by forwards and defenders [8]. Fullbacks, midfielders, and offensive midfielders were found to cover a greater high-speed distance, whilst central defenders and attackers did more high acceleration movements [33].

In the current body of work, center backs (CB) experienced the lowest absolute and relative physical demands. This finding is consistent with previous research investigating whole and peak match demands in elite soccer [15, 34]. This positional group was found to be less active in offensive transitions (ball possession) but achieved highest number of accelerations and decelerations – A+D (n · min^−1^) during defensive transitions (DT) (out of possession). It is noteworthy to acknowledge that accelerations and decelerations are more physically demanding than speed-based activities [35–36] and have been linked to match result in modern soccer [37]. Therefore, center backs should be regularly exposed to adequate accel/decel stimuli in training to be optimally prepared for high mechanical demands during collective defensive actions in matches. It was also revealed that this positional group’s locomotor metrics were raised when a team was out of ball possession [24]. In the current study, center backs (CB) along with central defensive midfielders (CDM) had the lowest SD (m · min^−1^), absolute SD, and A+D (n · min^−1^). Interestingly, these two positional groups showed very similar patterns for high-intensity metrics such as HSRD (m · min^−1^), SD (m · min^−1^), and A+D (n · min^−1^) during all transitions, suggesting similarities within parts of their training content. Similar training modes could be applied to best prepare them for high physical demands during transitional play, especially when the ball possession is lost and a rapid transition from offense to defense required.

In contrast, fullbacks (FB) covered the highest sprint distance in all transitions and near-maximum velocity actions. Predominantly displaying this during defensive transitions (DT) and fast attacks (FA), which is consistent with previous work [15, 24, 38]. It has been shown that maximum speed activities over longer distances were not adequately used in soccer training [39]. This should be considered when planning training for fullbacks. They often link defensive and offensive responsibilities, resulting in them accumulating high speeds over longer distances [13]. Prompting the need for them to be provided with regular high-velocity exposures during a weekly microcycle. It is important to remember high-velocity distances were found to be directly related to the training status [40] and recognized as one of the key indicators of the physical performance in professional soccer [41]. Fullbacks should be conditioned to cope with higher sprint distance exposure to best prepare them for competition. Allowing them to deliver high performance, meet their positional requirements and importantly keep them free from injury. Due to the fatiguing nature of these repeated high-velocity actions coaches should consider employing appropriate recovery strategies post training and game play. This is due to their link to increased injury risk [42–43]. Future research should consider analysing the effectiveness of recovery strategies post increased exposure to these high-velocity demands.

Additionally, central attacking midfielders (CAM) exhibited higher TD (m · min^−1^) and HSRD (m · min^−1^) than other positions. Interestingly, wingers (W) demonstrated the highest number of accelerations and decelerations per min – A+D (n · min^−1^) during offensive actions while in possession of the ball such as counterattacks and fast attacks. These findings are consistent with previous studies [15, 44]. These rapid changes of speed are crucial in elite soccer and the ability to accelerate, decelerate and change direction quickly has been linked to success in field-sport teams [3, 35] and soccer match result [37]. Interestingly, during counterattacks wingers (W) showed the highest output in other variables per minute (m · min^−1^) such as TD, HSRD and SD, which indicated the importance of this positional group during rapid changes from defensive to offensive transition. Previous literature detailed the significance of high-velocity metrics for creating opportunities and scoring goals, which has very strong implications on performance [17]. Counter-attacks demonstrated the need to generate high-velocity outputs to exploit opponents, open space and create goal scoring opportunities [45–46].

It has been shown that offensive midfielders covered the greatest locomotor distance per minute – TD (m · min^−1^) [38], while wide midfielders (wingers) accumulated the highest A+D (n · min^−1^) in 4-4-3 formation [24]. Findings consistent with the present study. Attackers (A) experienced lower physical output in defensive transitions (DT) but reached higher metrics during high pressure activities (HP), again consistent with conclusions drawn within literature [24, 44]. Activities that require players to re-gain possession, squeeze space and block penetrating forward passes, have been shown to generate a greater frequency of high-intensity accelerations and decelerations [14, 47]. These actions are very reactive to the opposition movement to apply immediate pressure on the ball. It has been shown that nearly half of all ball winning turnovers across different European leagues, were a result of high pressing in the offensive (opposition) half. Resulting in more goals scored [45, 48]. Thus, demonstrating how crucial it is to use attackers as the first line of defense, aiming to win the ball back and apply high pressure when out of possession.

Finally, mean transition performance for each playing position demonstrated higher match-to-match variability for absolute sprint distance (SD) than for relative sprint distance (Rel B5) (53.8% vs 32.7%, respectively). Indicating the high unpredictability of contemporary football matches and the complexity of modern training design for practitioners [49–50]. Global zones widely adopted in soccer enable work-load comparisons to be made between athletes, but don’t reflect players’ individual physical characteristics [3, 34, 50–51]. Relative speed zones could be more appropriate in elite soccer players analysis to assess true workload, detect fatigue and/or adaptation within a game [52] and therefore, should be explored more in future research.

Prescribing training based on whole/average match data [7], utilizing one physical variable (i.e. distance per min), while neglecting other contextual factors like position, limits specificity. Consequently, not fully reflecting the high physical stress players are exposed to in short and specific high-intensity passages within a soccer match [13, 15, 39, 53]. It is therefore critical for coaches to contextualize soccer-specific short-lasting blocks, that expose players to maximum physical outputs. Describing the tactical phase of play and positional role to best prepare athletes for competition, decrease the injury risk and improve performance [22]. Previous research has shown that utilising principles of overload of short-lasting maximum intensity activities within a training block, are beneficial to soccer players in impacting their performance and causing various physiological adaptations [2, 43, 54]. According to Bortnik et al. [14], if transitions were well described in relation to positional high-intensity and high-velocity metrics, they could offer new multivariate insights into the physical demands of the modern game [14]. Thus, allowing managers and fitness practitioners to plan a clearer contextualized plan for training prescription for the team and individual position [15]. To the authors knowledge, this is the first study to describe physical demands and positional differences in detail during the key phases of a soccer game (transitions).

The findings of this study should be used to inform training intensity to ensure that sessions reflect different physical requirements among varied positional groups. Utilising this approach within varying tactical drills (offensive and defensive) when in or out of possession. For instance, during the midweek overload block (MD-4 and/or MD-3), coaches could use specific drills which encompass transitional activities (defense-to-offense transitions, offense-to-defense transitions, and fast attacks) as well as transitional games (large-, medium-, and small-sided) to over-stimulate locomotor and mechanical demands, increasing the high-velocity stress placed on players [15, 39, 44]. Alternatively, practitioners could utilize different SSG’s to apply high pressure without the ball (defensive) and mimic high-intensity periods with the ball (offensive). By changing the number of players, size of the pitch and introducing a goalkeeper, the locomotor demands could be over- or under-stimulated relative to the WCS periods [38]. Large-sided games and small-sided games have been found to be the most appropriate mode of soccer-specific training (midweek) to over-stimulate different physical variables in relation to the 5- and 10-min peak match demands (125% sprint demands, and 150% high-intensity accelerations/decelerations, respectively) [38]. However, SSG’s could potentially under-stimulate locomotive variables such as distance, high speed, and sprinting [38]. Thus, emphasizing the importance of utilizing transitional activities to replicate the position-specific maximum physical demands experienced by the team or individuals during competition. This approach should be replicated within end-stage rehabilitation sessions to increase performance and reduce the risk of future injury. Importantly, these physical demands would change in relation to individual physical capacity, willingness to complete, coaching style, level of opposition, and other situational and contextual factors [49, 55–56].

Studies investigating the impact of contextual variables (match location, match half, and match outcome) on WCS within a soccer match are limited [24, 44, 57]. Contextual factors most likely determine the physical stress players are exposed to during the most demanding passages in modern match play, particularly for the high-velocity activities. This should be further explored [49]. The present body of work did not analyse effective playing time. Previous research has recently shown that physical demand increases with greater playing time (< 65 min) regardless of the playing position [58]. Our study analysed ten games (small sample) of only one club. Thus, future research should investigate a larger sample across a higher number of teams in a season. Also, consideration should be given to analysis of the impact of substitutions on physical metrics during TA’s, comparing training to game play and determine the impact of additional contextual factors such as formation, venue, score, to gain deeper knowledge about the true physical demands of the modern football game. The present study compared absolute to relative sprint distance across different positions during TA’s, but future research should also consider analysing peak intensity periods using acceleration thresholds to better relate to the players individual physical capacities during these actions.

## CONCLUSIONS

Understanding differences in physical absolute and relative outputs across different playing positions during peak intensity periods is important to practitioners. The present investigation shows that the key physical metrics are largely increased compared to the 90-min averages when contextualized into transitions. Also, differences exist in physical outputs across positions during these highest physical demands players are exposed to in match play. This study provides guidance for coaches and practitioners on how to prescribe short and specific high intensity activities in training that integrates physical and technical-tactical aspects (offensive and defensive) to mimic maximum physical demands of competition in relation to different positional groups. This knowledge has direct implications for training prescription in relation to high-intensity and high-velocity activities within a weekly microcycle to best prepare each positional group for peak intensity passages in football match.

### Key points summary

– Transitional activities largely increased physical demands across all positions compared to the 90-min averages.
– Coaches could use strategies that mimic these physical demands and replicate the positional specificity by designing appropriate drills in training, selecting offensive/defensive activities in/out of the ball possession, manipulating pitch size and the number of players to best prepare athletes for their position-specific role in the modern game and reduce their risk of injury (underpre-pardness).
– Center backs experienced the lowest physical metrics during all TA’s but achieved the highest number of high-intensity accelerations and decelerations in defensive transitions.
– Fullbacks covered the highest sprint distance during all transitions, which was most evident in defensive transitions and fast attacks.
– Central attacking midfielders ran higher total distance and high-speed running distance from other positions during offensive transitions (counter-attacks) and fast attacks.
– Wingers had the highest number of high-intensity accelerations and decelerations, especially during offensive activities (counter-attacks) and fast attacks.
– Attackers experienced lower physical demands during defensive activities, while reaching higher physical metrics during high pressure activities.
